# Unveiling the Scientific Landscape and Trends of Cutaneous Immune-Related Adverse Events: A Bibliometric Analysis

**DOI:** 10.7759/cureus.105013

**Published:** 2026-03-10

**Authors:** Danli Zhong, Chengyu Shi

**Affiliations:** 1 Department of Dermatovenereology, The Seventh Affiliated Hospital, Sun Yat-sen University, Shenzhen, CHN

**Keywords:** bibliometric analysis, ctla-4 blockade, cutaneous immune-related adverse events, immune checkpoint inhibitors, pd-1

## Abstract

With the widespread use of immune checkpoint inhibitors (ICIs) in cancer treatment, cutaneous immune-related adverse events (cirAEs) have attracted increasing attention. This bibliometric analysis reveals that research on cirAEs has grown steadily, with enhanced global collaboration. The focus has progressively shifted from early studies on specific agents and conditions toward severe cutaneous adverse reactions, diverse cancer types, and systemic clinical management strategies. This evolution reflects a deeper investigation into underlying mechanisms and a heightened emphasis on personalized treatment approaches, thus offering insights for future interdisciplinary research aimed at optimizing clinical management and improving treatment safety.

## Introduction and background

The advent of immune checkpoint inhibitors (ICIs) has revolutionized the treatment landscape for advanced solid tumors, demonstrating significant survival benefits in various malignancies, such as melanoma, non-small cell lung cancer (NSCLC ), and renal cell carcinoma. However, their non-specific immune activation can lead to immune-related adverse events (irAEs), with the skin and its appendages being the most frequently and earliest affected organs [1-3].

The incidence of cutaneous irAEs (cirAEs) ranges from 20% to 40%, with diverse clinical presentations [4]. These manifestations vary from mild pruritus and maculopapular rash to more severe conditions, such as psoriasiform eruptions, bullous pemphigoid, and even life-threatening severe cutaneous adverse reactions (SCARs) like Stevens-Johnson syndrome (SJS), toxic epidermal necrolysis (TEN), and drug reaction with eosinophilia and systemic symptoms (DRESS) [5,6]. These cutaneous toxicities not only cause physical discomfort and psychological burden for patients but also pose critical challenges in clinical decision-making regarding whether to pause or discontinue immunotherapy. If not managed promptly and appropriately, these reactions can become life-threatening [7].

Paradoxically, a growing body of evidence suggests that the occurrence of cirAEs may be positively correlated with improved ICI efficacy and patient survival, framing a complex "efficacy-toxicity paradox." Multiple retrospective and prospective studies have demonstrated this association. A seminal 2025 study involving a retrospective analysis of 278 patients with advanced solid tumors found that patients who developed cirAEs had a median progression-free survival of 47.3 months, significantly longer than the 18.3 months in the group without skin reactions. The median overall survival was extended to 60.0 months compared to 26.0 months in the control group, a statistically significant difference [8]. A multi-institutional cohort study found that cirAE development was associated with decreased mortality (HR=0.87), with particularly strong associations for specific morphologies, such as vitiligo (HR=0.29) and lichenoid eruptions (HR=0.51) in melanoma patients [9]. A large population-level analysis of over 14,000 patients confirmed that the development of cirAEs (including pruritus, drug eruption, and nonspecific rash) was significantly protective of mortality (HR=0.778 for any cirAE) [10]. This paradoxical link suggests that cirAEs could serve as clinical biomarkers for treatment response, yet the precise immunological mechanisms underlying this association and the heterogeneity among cirAE subtypes remain incompletely characterized.

Driven by these pressing clinical challenges and intriguing scientific questions, research in this area has proliferated. The volume of scientific literature on cirAEs has grown exponentially over the past decade, mirroring the expanding clinical use of ICIs. However, there remains a significant research gap: a lack of systematic bibliometric analysis to synthesize and elucidate the developmental patterns, knowledge structure, and collaborative networks in this rapidly evolving field. Bibliometrics, through the quantitative analysis of academic publications, can reveal research hotspots, trends, and intellectual landscapes, thereby guiding future research directions. While successfully applied in other dermatological fields such as atopic dermatitis [11] and psoriasis [12], a comprehensive bibliometric study has yet to be conducted for cirAEs.

Therefore, this study aims to fill this gap by performing the first comprehensive bibliometric analysis of the cirAEs field. We seek to quantify its exponential growth, map its developmental trajectory and knowledge architecture, identify research hotspots and emerging trends, and analyze international collaboration networks. Using data from the Web of Science Core Collection and analytical tools including CiteSpace, VOSviewer, and R Studio, we systematically examined publication trends, global collaboration networks, core journals, citation patterns, and keyword evolution. By providing a visualized, evidence-based framework, this study will help researchers understand the overall landscape, pinpoint key unresolved questions (e.g., the mechanisms of the efficacy, toxicity paradox, and strategies for personalized risk stratification), and identify potential collaborations. Ultimately, this analysis aims to facilitate scientific progress and contribute to the optimization of clinical management for cirAEs, improving outcomes for cancer patients navigating the complexities of immunotherapy.

## Review

Materials and methods

Data Source

The data were retrieved from the core collection of Web of Science (including Science Citation Index Expanded (SCI-Expanded) and Social Sciences Citation Index (SSCI)), a database widely recognized for its high authority and comprehensive coverage. As a well-established multidisciplinary citation database, Web of Science encompasses a broad range of high-impact journals spanning diverse scientific disciplines. It provides a comprehensive citation index, enabling in-depth exploration of the citation relationships among documents. Given that research on cirAEs involves multiple intersecting disciplines, the inclusion of a substantial number of journals from various fields in this database is particularly valuable. This ensures that the literature search on cirAEs is not limited to a single specialty, but rather incorporates relevant studies from several related domains, including immunology, dermatology, oncology, and pharmacology.

Search Strategy

To ensure the retrieval of all relevant literature on cirAEs, a comprehensive and precise search strategy was devised. The following search terms restricted to topic were employed: ("immune checkpoint inhibitor*" OR "immune checkpoint blockade*" OR "ICIs" OR "PD-1" OR "PD-L1" OR "CTLA-4" OR "nivolumab" OR "pembrolizumab" OR "ipilimumab") AND ("cutaneous immune-related adverse event*" OR "cutaneous adverse event*" OR "dermatological adverse event*" OR "skin immune-related adverse event*" OR "skin adverse event*" OR "cirAEs" OR "dermatological toxicities" OR "skin toxicity" OR "rash" OR "pruritus" OR "bullous pemphigoid" OR "vitiligo" OR "Stevens-Johnson syndrome" OR "toxic epidermal necrolysis" OR "DRESS"). The literature search encompassed publications from the inception of the database up to December 31, 2024, to capture the complete evolution of the field.

Inclusion and Exclusion Criteria

Studies were included if they were articles or reviews focusing on cirAEs associated with ICIs and published in English. Conference abstracts, editorials, comments, book chapters, case reports, and non-English publications were excluded.

Data Extraction

All the collected articles that met the inclusion criteria, along with their complete records and cited references, were saved in both plain text and BibTex formats for subsequent in-depth analysis.

Visualization Analysis

In this study, countries or regions, authors and institutions, contributing journals, and keywords were analyzed using the bibliometric package in R Studio (version 4.2.1; RStudio Team, Boston, MA) [13]. Default functions and settings within the package were used for descriptive statistics and visualizations. Additionally, CiteSpace (version 6.1) was used to conduct the clustering and burst detection for keywords [14]. Based on the network clustering theory in CiteSpace, a network module value (Q) greater than 0.3 indicates a significant network structure. An average silhouette value (S) greater than 0.5 suggests that the clustering result is reasonable. The parameters were set as follows: time slicing from 1990 to 2024 (one year per slice), term source from title/abstract/author keywords/KeyWords Plus, node type as “Keyword”, cosine similarity measure, and log-likelihood ratio for cluster labeling. VOSviewer software (version 1.6.18; Leiden University, The Netherlands) [15] was used to perform co-citation network analysis and collaboration analysis. For keyword co-occurrence, a minimum occurrence threshold of five was applied. For collaboration networks, all authors and institutions were analyzed. The visualization was normalized by association strength and optimized for clustering. Overall, both of the above software programs were used for visualization analysis to gain insights into the field of cirAEs and discover the research frontier using a considerable quantity of data.

Results

Publication Output and Growth Trends

**Figure 1:**
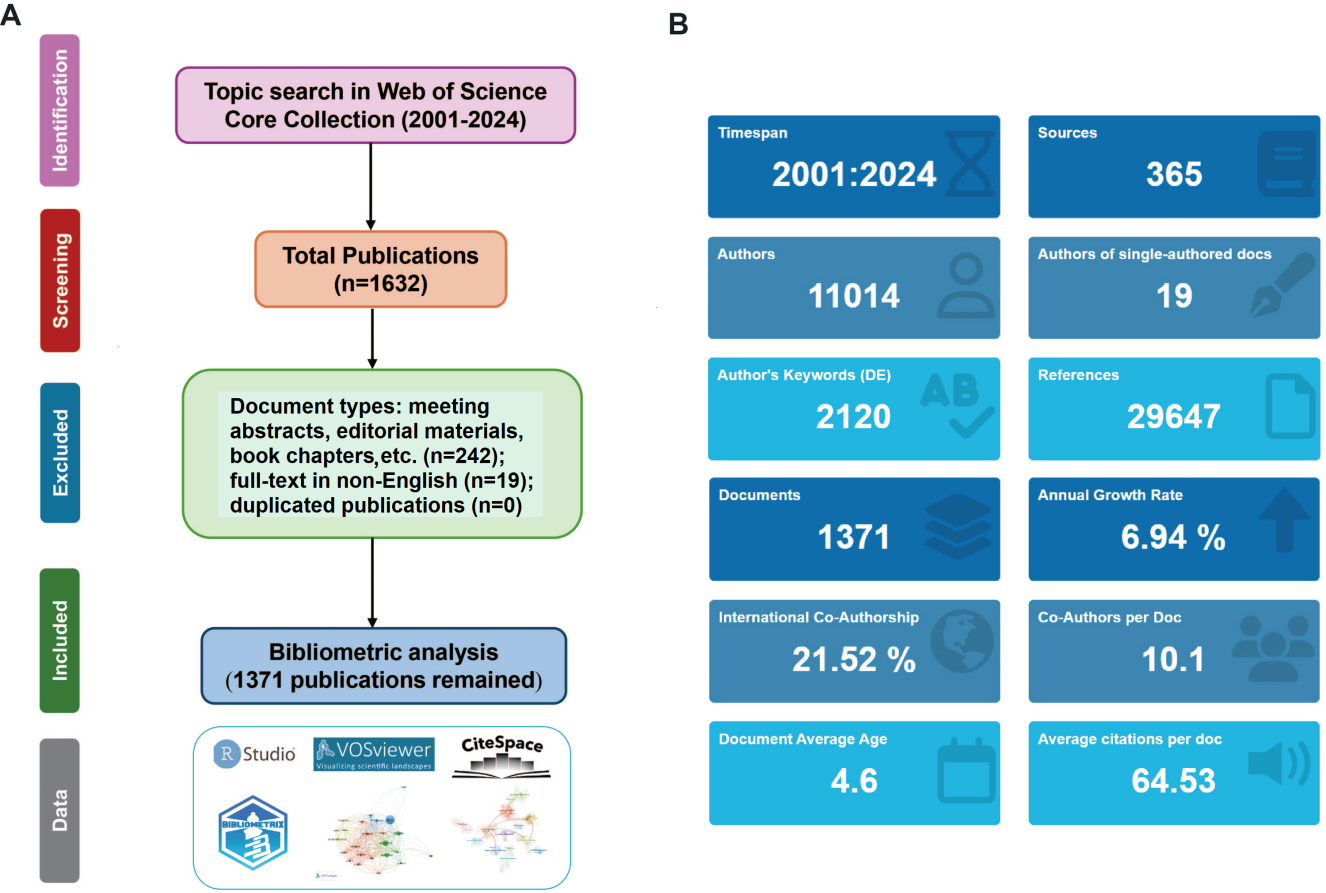
Flowchart of the study (A) A topic search in the Web of Science Core Collection (2001-2024) yielded 1,632 publications. After excluding 242 documents (document types: Meeting abstracts, Editorial materials, Book chapters, etc.) and 19 non-English full-text articles, 1,371 publications remained for analysis using tools (R Studio, VOSviewer, CiteSpace). (B) Key metrics of the included publications. All images were created by the authors using Adobe Illustrator.

**Figure 2:**
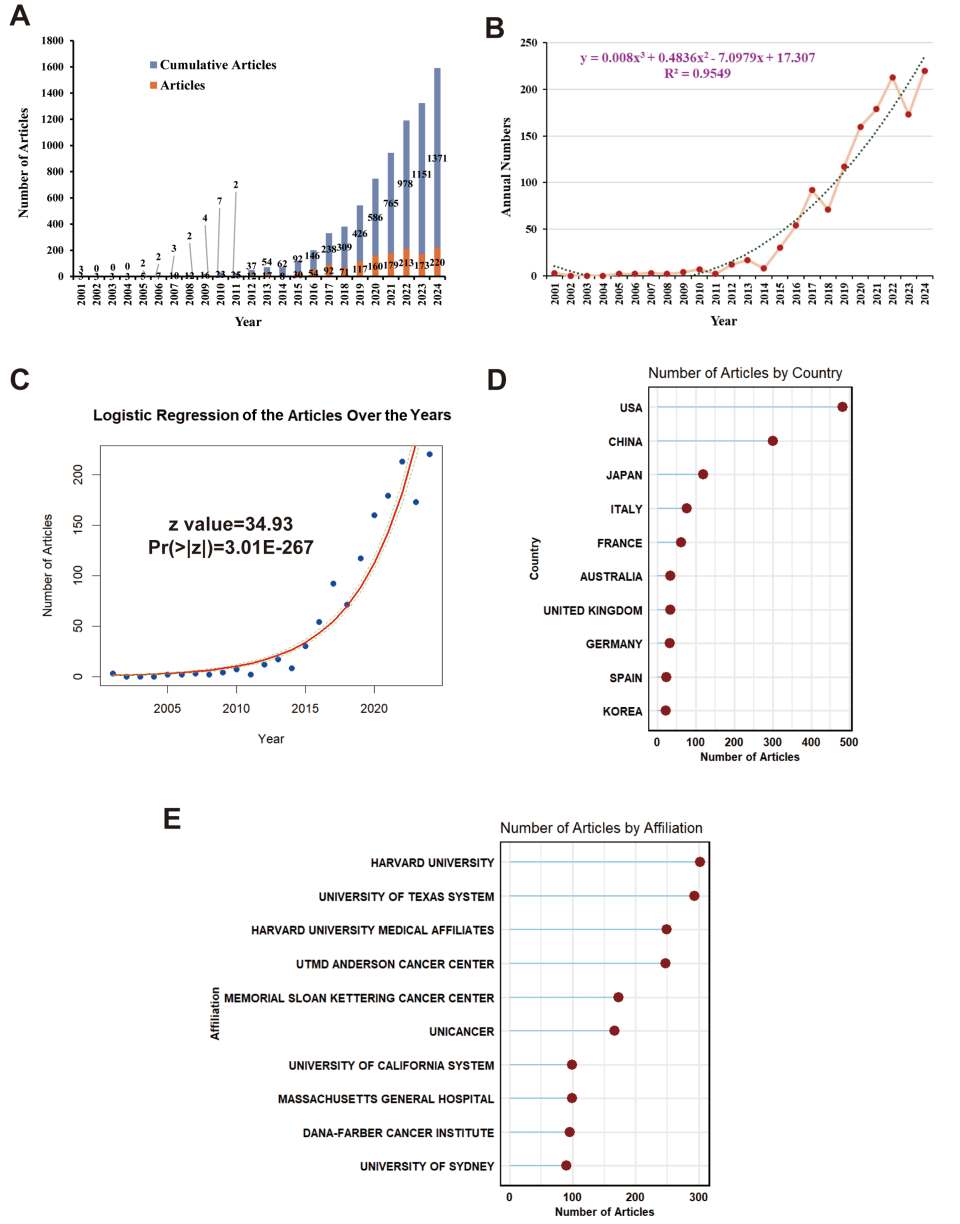
Publication trends and distribution (A) Annual number of articles (red bars) and cumulative articles (blue bars) from 2001 to 2024. (B) Fitted polynomial regression curve. (C) Logistic regression of articles over the years. Top 10 countries (D) and institutions (E) ranked by publication.

A total of 1,371 publications (1,095 original articles and 276 reviews) were included in the final analysis (Figure 1). The annual publication volume exhibited exponential growth, increasing approximately 76-fold from 2001 (n = 3) to 2024 (n = 230), with an estimated annual growth rate of 6.94% (Figure 2A). The growth pattern was further validated through polynomial and logistic regression analyses (y = 0.008X3 + 0.4836X2 - 7.0979X + 17.307, R2 = 0.9549, p = 3.01E-267, 95%CI: 0.240 (0.226, 0.253)), confirming a significant and reliable upward trend (Figures 2B, 2C).

Geographic and Institutional Contributions

The number of articles ranked by country (Figure 2D) and affiliation (Figure 2E) highlighted the contribution of different nations or research institutions to this research field. The United States had the most publications (482 articles) with joint publications in this discipline (multiple-country publications: 107), followed by China (300 articles) and Japan (119 articles). Six institutions produced more than 100 publications on cirAEs, with Harvard University leading the path with 302 publications (Table 1). 

**Table 1:** The number of publications of the top 10 countries and affiliations

Rank	Country (No. of Publications)	Affiliation (No. of Publications)
1	USA (213)	University of Texas MD Anderson Cancer Center (123)
2	China (86)	Harvard Medical School (83)
3	Italy (50)	Memorial Sloan Kettering Cancer Center (70)
4	Japan (37)	The Johns Hopkins University (47)
5	France (36)	Chinese Academy of Medical Sciences and Peking Union Medical College (39)
6	Germany (27)	Massachusetts General Hospital (36)
7	Australia (17)	Northwestern University (31)
8	Switzerland (17)	Vanderbilt University (29)
9	United Kingdom (17)	Dana-Farber Cancer Institute (27)
10	Greece (14)	Sapienza University of Rome (23)

Disciplinary and Journal Networks

**Figure 3:**
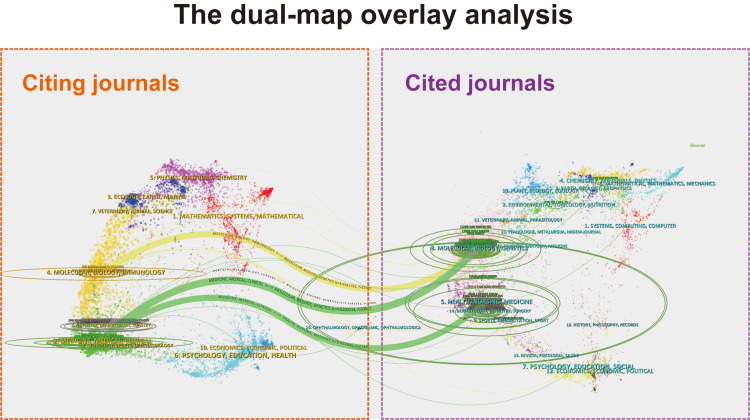
The dual-map overlay and corresponding disciplines The citing journals were on the left, the cited journals were on the right, and the colored path represents the citation relationship.

We employed dual-map overlay analysis to visualize the citation relationships between journals and to reveal interdisciplinary crossover (Figure 3). The left side of the map represents the citing journals. The three thickest citation trajectories indicate the predominant flows of research. The yellow trajectory demonstrates that articles published in molecular/biology/immunology journals were frequently cited by researchers in genetics journals. The green trajectory indicates that publications in medicine/medical/clinical journals were most commonly cited in health/nursing/medicine and molecular/biology/genetics journals.

Keyword and Topic Evolution

**Figure 4:**
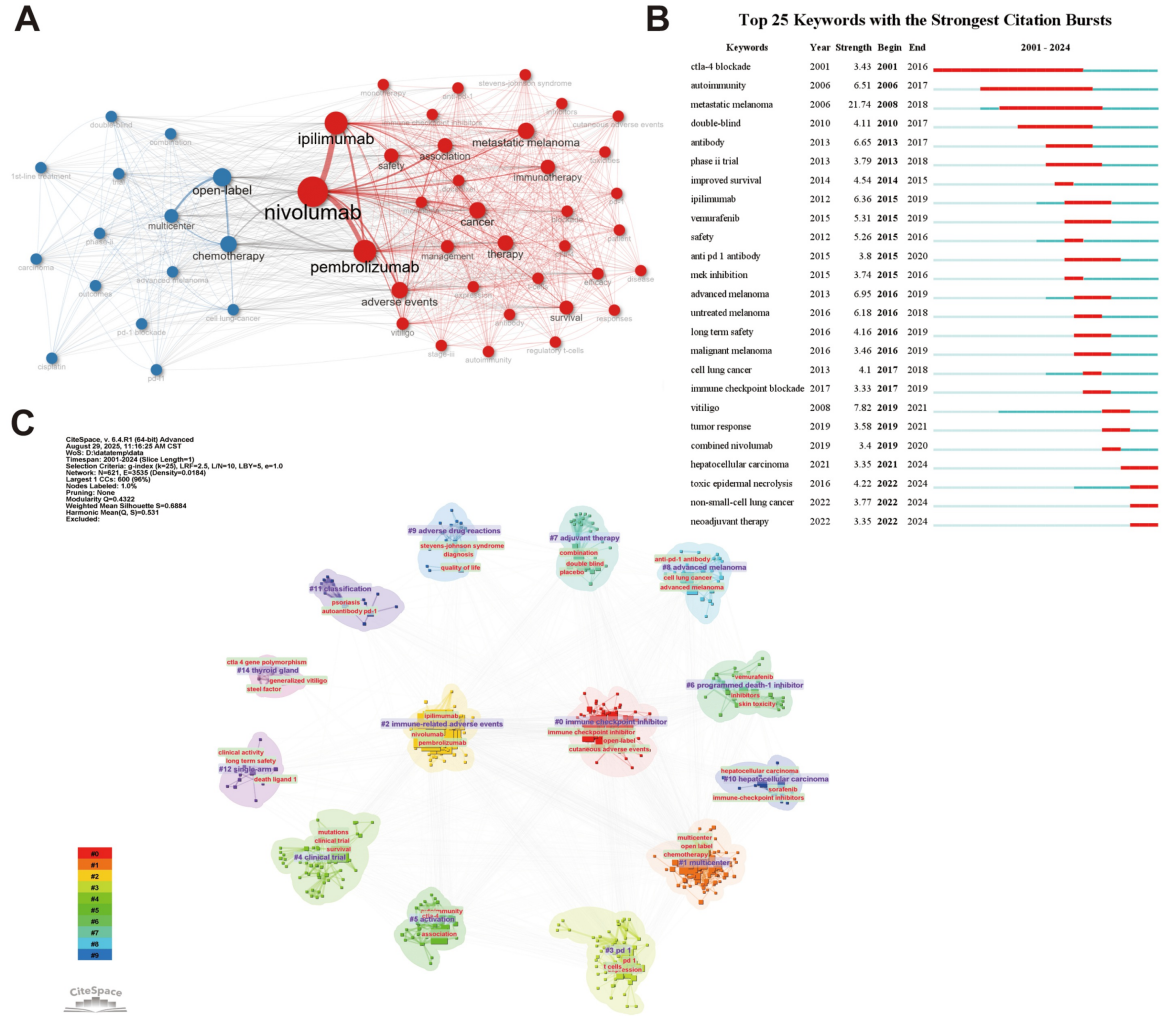
Visualized analysis of keywords and literature related to cutaneous immune-related adverse events (cirAEs) (A) The terms co-occurrence network of high-frequency keywords. Nodes represent keywords (top 50). Lines refer to the co-occurring relationship. Different colors represent two clusters. (B) Burst strength and time duration of the top 25 keywords with the strongest citation bursts. (C) Keyword clustering visualization map.

Keyword co-occurrence analysis was conducted to identify current research topics and map the scientific knowledge structure within the field. Our analysis of the top 50 keywords by frequency revealed a network (Figure 4A) centered around principal ICI agents, namely, "nivolumab", "pembrolizumab", and "ipilimumab". Additionally, the right side of the chart highlights primary oncological indications for ICI therapy (e.g., "metastatic melanoma", "cancer") and emphasizes dermatologic safety profiles (e.g., "adverse events", "safety", "cutaneous adverse events"). A distinct peripheral blue cluster - characterized by terms such as "chemotherapy", "open-label", "multicenter", and "1st-line treatment" - delineates studies comparing the efficacy and safety of ICIs to conventional chemotherapies, with the associated clinical trial methodologies.

A burst analysis of the keywords (Figure 4B) showed that research on cirAEs initially concentrated on immune checkpoint drugs, such as CTLA-4 blockade and specific antibodies, at the time of emergence. Over time, there was a shift towards paying more attention to types of cutaneous adverse reactions (e.g., TEN, vitiligo) and expanding to diverse cancer types (including hepatocellular carcinoma and non-small-cell lung cancer). This progression reflects the field's evolution from drug exploration to a comprehensive emphasis on safety and broader clinical applications.

The clustering map results (Figure 4C) indicated the following keyword clustering labels: Cluster #0 (immune checkpoint inhibitor), Cluster #1 (multicenter), Cluster #2 (immune-related adverse events), Cluster #3 (PD1), Cluster #4 (clinical trial), Cluster #5 (activation), Cluster #6 (programmed death-1 inhibitor), Cluster #7 (adjuvant therapy), Cluster #8 (advanced melanoma), Cluster #9 (adverse drug reactions), Cluster #10 (hepatocellular carcinoma), Cluster #11 (classification), Cluster #12 (single-arm), and Cluster #14 (thyroid gland). Clusters #1, #4, and #12 indicate that clinical research, especially large-scale, multi-site studies, is a crucial approach in this field. Clusters #0, #2, #3, and #9 focus on ICIs and their associated skin-related adverse effects, while Clusters #8 and #10 correspond to specific tumor types treated by ICIs. Finally, Cluster #7 suggests that the research extends to the role of auxiliary treatment methods in the context of immune checkpoint inhibitor use and skin adverse reactions.

Collaboration Network Analysis

**Figure 5:**
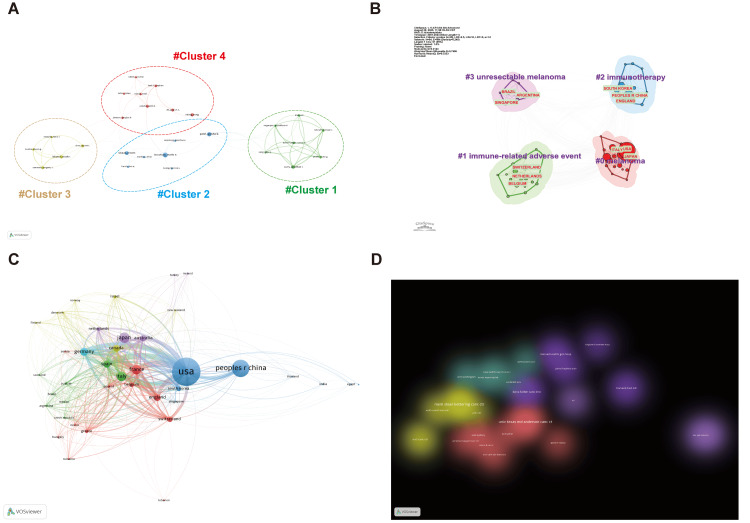
The collaborative network of countries/regions, affiliations, and authors (A) Collaborative network of the top 27 authors. (B) Collaborative network of 64 most productive countries/regions clustered by keywords. (C) The total link strength and documents of cooperation among the productive countries/regions. (C) Collaboration network of the top 23 affiliations.

Effective cooperation between institutions and countries plays a crucial role in promoting academic exchange and advancing scientific research. The collaborative networks of the top 27 authors form four distinct clusters (Figure 5A). While there are some connections between clusters of different colors, cross-cluster collaborations remain relatively limited. The clustered country collaboration network also comprises four clusters: melanoma, immunotherapy, unresectable melanoma, and irAEs (Figure 5B). The USA, Japan, and Italy play prominent roles in melanoma research. South Korea, China, and England are key contributors to immunotherapy. Brazil, Argentina, and Singapore are active in the unresectable melanoma cluster. Switzerland, the Netherlands, and Belgium are involved in the irAE cluster. Figure 5C illustrates the top 40 countries and regions with the highest number of publications in this field from 2001 to 2024. Among them, the USA and China are the two leading producers of research, and their collaboration significantly surpasses that of other countries. Additionally, the USA maintains relatively frequent cooperation with countries such as France, England, Australia, and Switzerland.

Citation Analysis 

**Figure 6:**
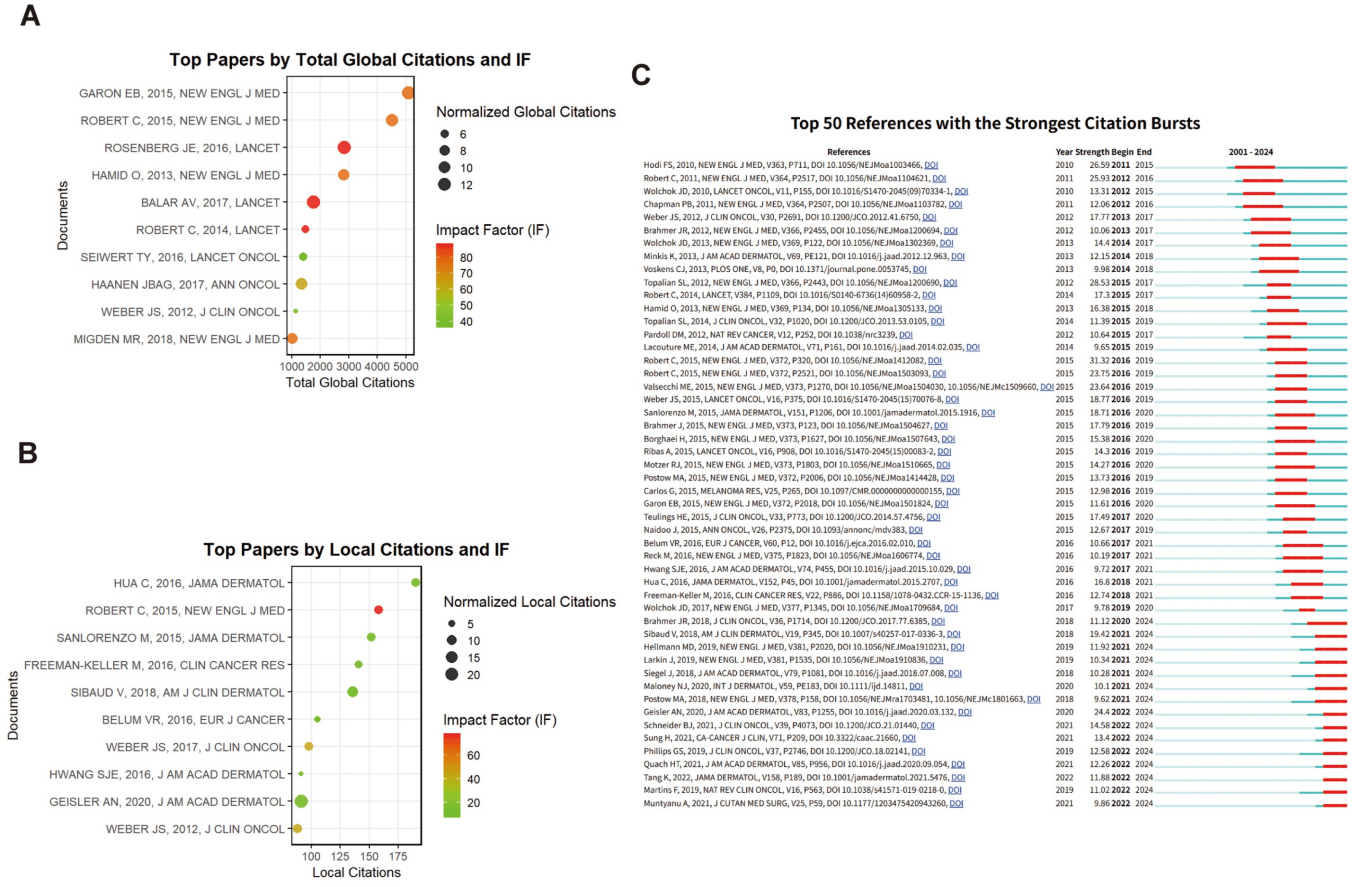
Citation analysis (A) List of the top 10 most globally cited documents on cirAEs, ranked by their global citation counts. Each document is labeled with the author(s) names, publication year, and journal. (B) List of the top 10 most locally cited documents on cirAEs, ranked by their local citation counts. Each document is labeled with the author(s) names, publication year, and journal. (C) Top 50 references with the strongest citation bursts.

Figure 6A presents global citation counts for articles in our dataset, reflecting their total citations in the Web of Science database. The most globally cited article (Garon et al., published in the New England Journal of Medicine) has been cited 5,088 times and reported pruritus as a common side effect of pembrolizumab in NSCLC [16]. The second most cited (published in the New England Journal of Medicine by Robert et al.) described fatigue, pruritus, and nausea as frequent adverse events with nivolumab [17].

A local citation analysis (Figure 6B) was conducted to identify the works most frequently cited within the analyzed dataset, reflecting their influence on cirAEs. The most locally cited article (Hua et al., JAMA Dermatology, with 190 citations) reported 25% patients developed vitiligo in those with metastatic melanoma treated with pembrolizumab [18].

Figure 6C displays the top 50 references with the strongest citation bursts. The article with the highest burst strength between 2016 and 2019 demonstrated superior survival outcomes with nivolumab versus dacarbazine in BRAF-wild-type melanoma [17].

Co-citation Analysis

**Figure 7:**
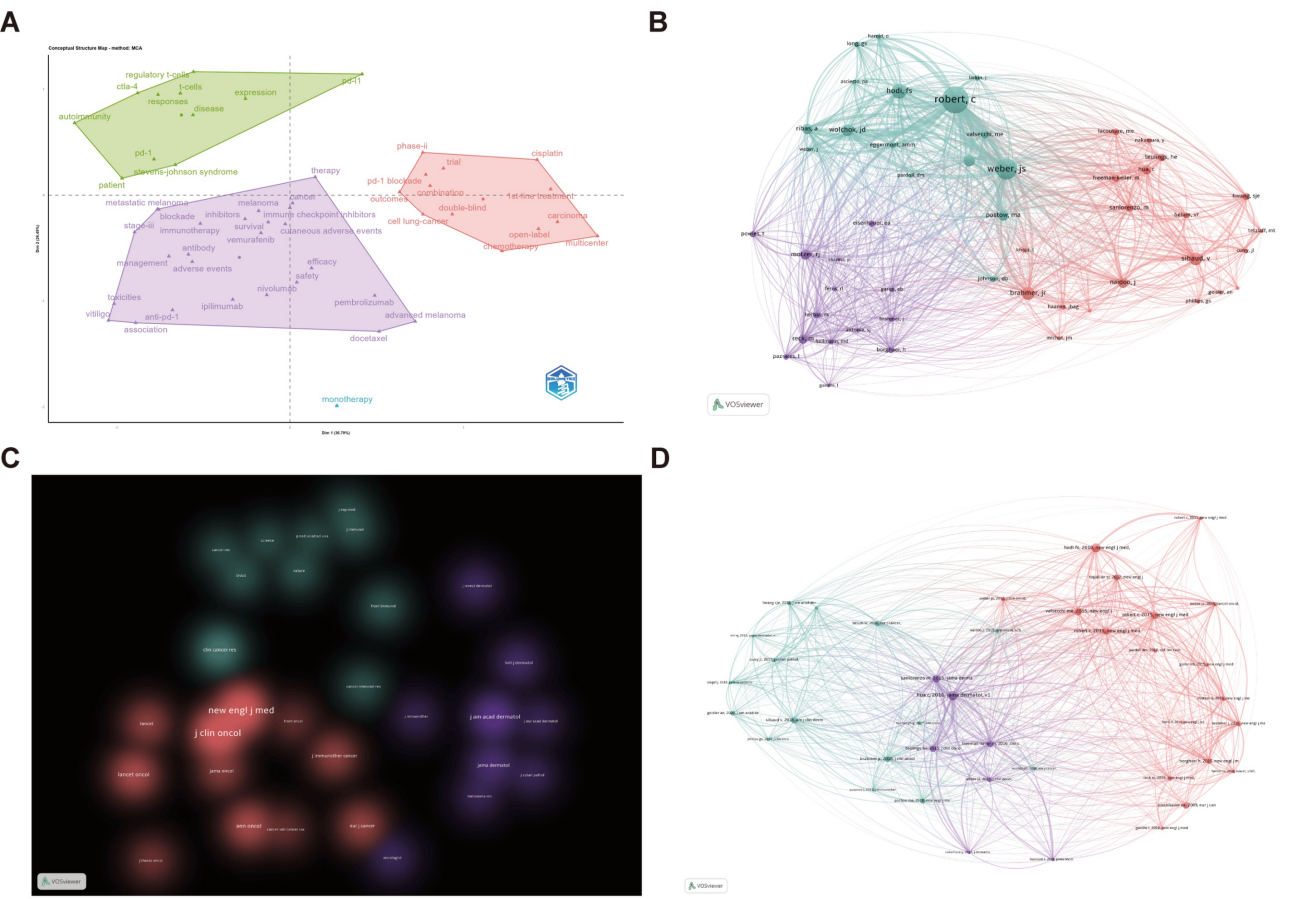
Co-citation network (A) Conceptual structure map of KeyWord Plus. The center of the map represents the center of the research topic and shows the main topics. Each color represents a different sub-factor. (B) Author co-citation network. (C) Journal co-citation network. (D) Literature co-citation network.

Two papers cited together by another document are considered to have a co-citation relationship. Unlike global citation analysis, co-citation networks emphasize research topics closely associated with specific fields. We used a multidimensional scaling method to classify the most frequently occurring keywords and generate a conceptual structure map. Keyword clustering (Figure 7A) revealed three main themes: clinical focus on disease types, interventions, and prognosis (purple); pathogenesis and molecular mechanisms (green); and comparative efficacy/safety of ICIs versus chemotherapy with associated clinical trial methodologies (red).

Co-author analysis (Figure 7B) highlighted three researcher clusters: oncology/immunology experts such as Robert C. and Weber J.S. (teal), dermatology-focused investigators like Sibaud V. and Hua C. (red), and interdisciplinary contributors bridging oncology and immunology such as Motzer R.J. (purple). Central authors in each cluster demonstrate dense co-citation links, reflecting their influence and collaborative roles.

Journal co-citation (Figure 7C) further illustrated interdisciplinary integration, with clusters representing high-impact oncology journals (e.g., New Engl J Med and J Clin Oncol, central to the red cluster), dermatology-specific publications (e.g., J Am Acad Dermatol and JAMA Dermatology, corresponding to the purple cluster), and broad-scope scientific (including the teal one with Nature and Science) or immunology-focused periodicals (e.g., J Immunol and Front Immunol).

Document co-citation (Figure 7D) grouped influential literature into mechanistic foundations (green), clinical management studies (red), and translational research (purple), with cross-cluster citations indicating integration across domains.

Together, these analyses underscore the interdisciplinary nature of cirAEs research, driven by collaboration across oncology, dermatology, and immunology. Co-citation analysis revealed thematic and collaborative structures within the field.

Research Hotspots in cirAEs

**Figure 8:**
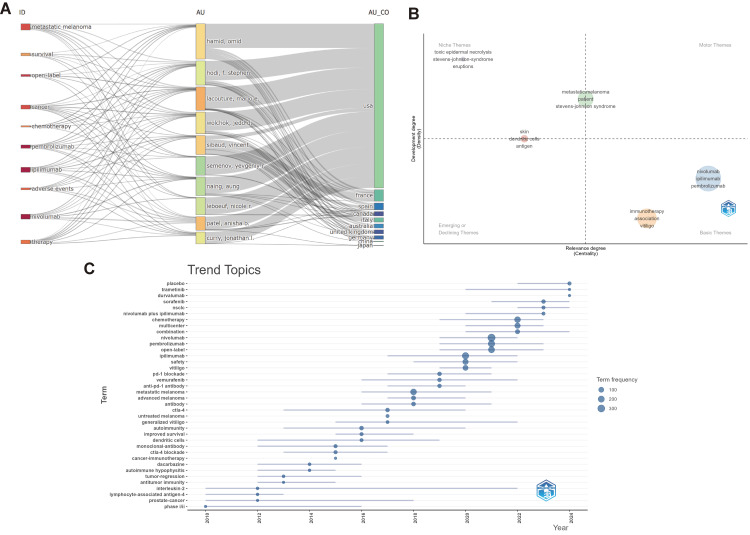
Evolution of research related to cutaneous immune-related adverse events (cirAEs) (A) Three-field plot of the keywords analysis in the field of cirAEs (left field: authors; middle field: keywords plus; right field: institution). (B) Thematic analysis in the field of cirAEs. The horizontal and vertical axes represent centrality and density, respectively. The first quadrant is well-developed topics, the second quadrant is not important to the current field, the third quadrant is topics that may have recently emerged or may soon disappear, and the fourth quadrant is basic topics that are not important. (C) Timeline of research trends.

Figure 8A presents the relationships and distribution patterns among the top 10 research authors (left), topics (middle), and institutions (right) in the field of cirAEs. While most authors and institutions have contributed to these key areas, significant differences exist. Authors such as Curry, Jonathan I., and Hamid Omid focus on topics including “nivolumab”, “ipilimumab”, and “adverse events”. The University of Texas System demonstrated a stronger emphasis on "chemotherapy", "nivolumab", and "scaffolds", whereas Harvard University focused primarily on "survival" and "adverse events". Figure 8B presents nivolumab, ipilimumab, and pembrolizumab, located in the "Basic Themes" quadrant, showing high relevance and forming the fundamental focus of the field. "Immunotherapy" and eruption types, such as "vitiligo" in the same quadrant, also play important roles. "Metastatic melanoma" and "Stevens-Johnson syndrome" in the middle area have moderate relevance and development degree. "Toxic epidermal necrolysis", "Stevens-Johnson syndrome", and "eruptions" in the "Niche Themes" quadrant have a certain development degree but relatively lower relevance. "Skin", "dendritic cells", and "antigen" in the "Emerging or Declining Themes" quadrant seem to have limited current influence. Overall, ICIs (e.g., nivolumab) are core basic themes, while niche themes related to specific severe skin reactions also exist, and the field has a mix of well-established and developing topics. This trend topics graph (Figure 8C) showcases the evolution of research themes in cirAEs over the past 23 years. In recent years, terms such as "placebo", "trametinib", and "durvalumab" have gained prominence, indicating growing interest in new agents and control groups. Combination therapies, such as "nivolumab plus ipilimumab", also show increasing attention. Core ICIs, such as "nivolumab" and "pembrolizumab", maintain high term frequencies, reflecting their sustained central role. Earlier themes, such as "dendritic cells" and "cancer immunotherapy", had relevance but now appear less prominent compared to newer topics. Terms related to specific skin conditions (e.g., "vitiligo") and treatment safety remain steadily discussed. Overall, the field has shifted toward novel therapies, combination approaches, and refined exploration of adverse reactions while retaining focus on key inhibitors.

Discussion

Principal Findings

This study presents a systematic bibliometric analysis of 1,371 global publications on cirAEs from 2001 to 2024 using bibliometric methods. To our knowledge, this is the first comprehensive bibliometric review to map the developmental trajectory, collaborative networks, and shifts in the research focus within the field of cirAEs associated with ICIs. Our findings delineate the intellectual structure of this rapidly evolving domain and highlight its intrinsic link to the clinical translation of cancer immunotherapy.

The exponential increase in publication output - 76 times greater in 2024 than in 2001 - closely parallels the expanding clinical use of ICI applications. This trend reflects how cirAEs have progressed from sporadic case reports to a structured research area, driven largely by the widespread adoption of PD-1/PD-L1 and CTLA-4 inhibitors as first-line therapies for malignancies, such as melanoma and NSCLC [19]. The observed logistic growth pattern further confirms that cirAEs research has entered a phase of accelerated development, aligning with global trends in immuno-oncology.

Several factors underpin this growth. First, the increasing clinical application of ICIs has led to a higher incidence of cirAEs, thereby stimulating research into their mechanisms, diagnosis, and management. As PD-1/PD-L1 inhibitors (e.g., nivolumab, pembrolizumab) and CTLA-4 inhibitors (e.g., ipilimumab) have become standard treatments for cancers such as melanoma and NSCLC, the clinical impact of cirAEs, including reduced patients' quality of life, treatment interruption, and life-threatening events, has become more apparent, driving academic inquiry. Second, advances in diagnostic techniques and heightened clinical awareness have further accelerated research progress. Improvements in dermoscopy, histopathology, and molecular detection have enabled more precise classification of cirAEs [20-22], including conditions such as vitiligo, SJS, and TEN, thereby laying a foundation for mechanistic and clinical studies. Meanwhile, increased clinical vigilance has led to more frequent reporting of mild or rare cases, enriching the available data [23,24]. This trend closely mirrors the global evolution of cancer immunotherapy, indicating that cirAEs research has become integral to the clinical translation of ICIs.

Evolution of Research Themes

Dual-map overlay and keyword analyses revealed a distinctly interdisciplinary research landscape. Core citation paths connecting molecular/biology/immunology with medicine/clinical and genetics underscore the necessity of integrating expertise across oncology, dermatology, immunology, and genetics.

Keyword co-occurrence and burst analyses further traced how research hotspots have evolved alongside clinical needs. The early phase (2001-2010) was characterized by drug exploration and case reports of CTLA-4 inhibitor-related toxicities [25,26]. Following the approval of PD-1/PD-L1 inhibitors (from 2011 to 2020), the focus shifted toward mechanistic investigation and diagnostic optimization [27,28]. For example, studies have linked regulatory T cell (Treg) dysfunction to the development of irAEs, highlighting the need for multidisciplinary collaboration to unravel underlying mechanisms. Immunologically, Tregs (CD4+FoxP3+) are essential for maintaining immune tolerance. Their impairment can lead to overactivation of autoreactive T cells and abnormalities in helper and memory T cells, as observed in tissues affected by irAEs [29,30]. Specifically, instability in FoxP3 expression may drive Treg conversion into pathogenic effector cells, directly contributing to autoimmune responses [31].

In the most recent period (2021-2024), research has increasingly emphasized biomarker-driven risk stratification and the management of combination ICI regimens [32-34].

Clinical and Translational Implications

Beyond quantifying growth, our keyword and burst analyses identified a notable thematic shift. While early research logically centered on drug-specific toxicities, the field has increasingly focused on understanding severe, life-threatening cirAEs and their occurrence across diverse cancer types. This evolution mirrors the clinical trajectory: from initial efficacy validation to managing complex toxicities in broader patient populations.

Critically, this shift coincides with a fundamental re-evaluation of pathogenic paradigms, which provides a scientific framework for interpreting the well-documented yet "paradoxical association" between cirAEs and improved treatment outcomes [35-37]. The paradox lies in the fact that adverse events, indicative of immune system overactivation against self-tissues, are statistically linked to superior anti-tumor efficacy. Our bibliometric map positions this association as a key contemporary research nexus, bridging mechanistic studies with translational questions.

Recent high-impact studies signal a move from descriptive phenomenology towards deciphering shared and distinct immunologic pathways that may underpin this paradox [38,39]. For instance, a 2025 study challenged the traditional Th1/Th2 dichotomy in cirAEs, identifying shared Th2 immune features in both ICI-induced lichenoid and eczematous eruptions [40]. This finding suggests that the immune deviation in cirAEs may be more nuanced than previously thought and that certain Th2-skewed responses, perhaps through engaging specific cytokine networks or tissue-resident memory T cells, could be concurrently facilitating both skin inflammation and productive anti-tumor immunity. This re-evaluation implies that the mere occurrence of inflammation may be less predictive of efficacy than its specific qualitative character.

Concurrently, mechanistic research has pinpointed specific molecular players that could explain the localized severity of some cirAEs and their potential link to systemic anti-tumor response [41,42]. A landmark study utilizing single-cell RNA sequencing revealed that severe cirAEs, such as SJS/TEN, are driven by macrophage overexpression of the chemokine CXCL10 [43]. CXCL10 is a potent chemoattractant for cytotoxic CD8+ T cells, leading to their excessive clustering in the skin and resultant tissue damage. This mechanism is highly instructive for the paradox: CXCL10 is also a key chemokine induced by interferon-gamma (IFN-γ) in the tumor microenvironment, crucial for recruiting anti-tumor CD8+ T cells. Therefore, the systemic immune activation by ICIs that successfully generates a CXCL10-high, T-cell-inflamed tumor microenvironment might spill over, via circulating factors or shared antigenicity (as seen in vitiligo associated with melanoma), to similarly inflame the skin. This creates a biological plausibility for cirAEs as a bystander marker of a robust, systemically activated, and potentially effective anti-tumor immune response. The therapeutic corollary - using anti-TNF biologics instead of broad steroids to manage such severe reactions - aims to dampen the damaging inflammation while potentially preserving the beneficial anti-tumor immunity, directly addressing the clinical dilemma at the heart of the paradox [44].

Interdisciplinary Integration

The interdisciplinary synergy has been essential in driving the transition from describing adverse events to understanding their immunopathology and interpreting the efficacy paradox. This progression is exemplified by the integration of dermatology, oncology, and immunology to study tissue-specific immune dynamics. Despite this progress, the precise mechanisms linking cirAEs to favorable antitumor responses remain incompletely characterized. Given the paucity of dedicated mechanistic studies in cirAEs, relevant hypotheses may be reasonably extrapolated from emerging evidence in other irAE organ systems.

Accumulating evidence implicates tissue-resident memory T cells (TRMs) as key cellular mediators of the efficacy-toxicity paradox. Utilizing spatial transcriptomics and multiplex immunofluorescence, recent studies have demonstrated that cirAE lesions - particularly ICI‑associated dermatitis - are predominantly infiltrated by CD4⁺ and CD8⁺ TRMs exhibiting a Th1/cytotoxic T cell 1 phenotype, with prominent expression of IFN‑γ, CXCL9, CXCL10, and TNF‑α [45-47]. Unlike circulating effector T cells, TRMs persist long term within peripheral tissues and are positioned to serve as local effectors of cross‑reactive autoimmunity. Their activation during ICI therapy likely reflects a systemic “unleashing” of pre‑existing tissue‑surveying T‑cell clones, a subset of which may possess tumor‑reactive specificities. Consequently, the clinical manifestation of cirAEs may represent a visible, cutaneous correlate of successful systemic T‑cell activation and tissue infiltration - a mechanism potentially shared with the intratumoral immune response. This paradigm underscores an urgent need for multidisciplinary collaboration among immunologists, dermatopathologists, and oncologists to spatially map and comparatively analyze immune infiltrates across tumor and affected normal tissues, thereby dissecting shared versus distinct immunologic programs.

Parallel and complementary lines of investigation have established Treg dysfunction as a central event in the pathogenesis of cirAEs [42]. From an immunological standpoint, Tregs are indispensable for the maintenance of peripheral tolerance; their functional impairment - whether quantitative or qualitative - can precipitate overactivation of autoreactive T cells, a finding consistently observed in irAE‑affected tissues. Clinically, ICI therapy potentiates antitumor immunity by alleviating Treg‑mediated suppression, yet this concurrently disrupts immune homeostasis and predisposes patients to multi‑system irAEs [28]. From a genetic perspective, genome‑wide association studies have begun to delineate causal relationships between Treg‑related immune signatures and susceptibility to autoimmune diseases [48]. Furthermore, single‑cell sequencing technologies have uncovered previously unrecognized Treg subsets with distinct transcriptional programs that may contribute to irAE development [30,49-51]. Therapeutically, strategies aimed at preserving Treg stability - such as low‑dose IL‑2 administration [52,53] or adoptive Treg cell therapy [54] - represent promising investigational avenues. Collectively, these multidimensional insights reinforce the critical importance of integrating immunology, clinical investigation, and genetics to unravel the complex, context‑dependent roles of Tregs in ICI‑induced toxicities.

The mechanistic frameworks centered on TRMs and Tregs are not mutually exclusive; rather, they likely operate in concert, reflecting the heterogeneous and patient‑specific nature of cirAEs. TRMs may serve as local amplifiers of tissue inflammation, while Treg dysfunction establishes a permissive environment for their expansion and persistence. Deciphering how these two cellular compartments interact - and how their interplay varies across skin subtypes, ICI regimens, and tumor types - will require coordinated, interdisciplinary efforts. Integrating high‑resolution spatial omics with functional validation in preclinical models and well‑annotated patient cohorts represents a logical next step. Ultimately, such mechanistic clarity is essential not only for developing targeted interventions to uncouple toxicity from efficacy but also for refining biomarker‑driven risk stratification and personalizing ICI therapy.

Interdisciplinary integration is also reflected in geographical and institutional collaborations. The United States ranked first in publication output, and its advantages stem from early deployment and interdisciplinary resource integration in cirAEs. China ranked second, reflecting the rapid improvement of its ICI clinical application and research capabilities in recent years. However, there remains considerable scope for advancement in mechanistic research and multi-center collaborative studies within this field.

Harvard University became the core institution, and its advantages lie in interdisciplinary platforms and clinical resource integration. This suggests that collaboration, including data sharing and joint clinical trials among institutions, can further improve research efficiency. Especially for studies on rare cirAEs, inter-institutional collaboration is the key to breaking through this bottleneck.

Globally and locally, highly cited literature all have the characteristic of "clinical orientation" [16] and [18]. Citation burst analysis further reveals emerging interest in refining therapeutic strategies for genetically defined subpopulations and offering evidence to support clinical safety evaluation [17]. These high-impact studies suggest that solving practical clinical problems is the core innovation direction of the cirAEs field.

Comparison With Previous Bibliometric Studies

Compared with previous bibliometric studies, earlier research has extensively covered the overall spectrum of irAEs [3,55], with particular attention paid to rheumatic [56] and endocrine [57] manifestations. To our knowledge, this study represents the first bibliometric analysis focusing specifically on cirAEs. Multiple bibliometric analyses consistently indicate that the top three countries in terms of publication output in the irAEs field are the United States, Japan, and China, while our findings show that the top three countries for cirAEs are the United States, Japan, and Italy. Regarding journal distribution, Journal for Immunotherapy of Cancer ranks as the journal publishing the highest number of studies on irAEs [3,55]. The research focus has gradually shifted from early case reports toward clinical trials and systematic reviews, reflecting the growing methodological maturity of the field. High-frequency keywords include "immune-related adverse events", "immune checkpoint inhibitors", "immunotherapy", and "nivolumab". Related studies cover various cancer types, such as melanoma, NSCLC, hepatocellular carcinoma, and renal cell carcinoma. Notably, while a wide range of adverse events has been reported, several organ-specific toxicities have gradually evolved into distinct research subfields, including endocrine toxicities, rheumatic and immune-related toxicities, pulmonary toxicities, hematologic toxicities, as well as drug utilization patterns and toxicity profiling.

Limitations

Our study has several limitations. First, reliance solely on Web of Science Core Collection may introduce database selection bias, potentially omitting relevant studies indexed in Scopus, MEDLINE, or Embase. Second, excluding non-English literature might underrepresent research contributions from certain regions, affecting the global landscape portrayal. Third, our assessment of collaboration based on co-authorship counts does not capture the quality or intensity of collaborative relationships. Finally, bibliometric analysis reflects publication trends but cannot directly assess the clinical impact or scientific quality of individual studies.

## Conclusions

This bibliometric analysis reveals the rapid growth of the cirAE research field from 2001 to 2024, driven by clinical needs and a successful interdisciplinary model. This model, best exemplified by leading institutions such as Harvard University, transcends simple collaboration. It embodies a disciplined pluralism that systematically integrates dermatology, oncology, immunology, and genetics to transform clinical observations into mechanistic insights, such as the roles of TRM cells and Treg dysfunction in the efficacy-toxicity paradox.

To address future global challenges, a differentiated research strategy is essential. Established leaders should leverage their deep interdisciplinary foundations to set standards and pioneer frontier mechanistic research. Ultimately, progressing from “multidisciplinary consultation” to institutionalized “interdisciplinary co-creation” will be key to advancing personalized prevention and supporting the safe, effective global use of ICIs.
